# NaoXinTong Inhibits the Development of Diabetic Retinopathy in *db*/*db* Mice

**DOI:** 10.1155/2015/242517

**Published:** 2015-03-03

**Authors:** Mengyang Liu, Quan Pan, Yuanli Chen, Xiaoxiao Yang, Buchang Zhao, Lifu Jia, Yan Zhu, Jihong Han, Xiaoju Li, Yajun Duan

**Affiliations:** ^1^State Key Laboratory of Medicinal Chemical Biology, Nankai University, 94 Weijin Road, Tianjin 300071, China; ^2^College of Life Sciences, Nankai University, 94 Weijin Road, Tianjin 300071, China; ^3^College of Medicine, Nankai University, 94 Weijin Road, Tianjin 300071, China; ^4^Collaborative Innovation Center for Biotherapy, Nankai University, 94 Weijin Road, Tianjin 300071, China; ^5^Buchang Pharmaceutical Co. Ltd., 50 Gaoxin Road, Xi'an 712000, China; ^6^Tianjin State Key Laboratory of Modern Chinese Medicine, Tianjin University of Traditional Chinese Medicine, 312 Anshan West Road, Tianjin 300193, China

## Abstract

Buchang NaoXinTong capsule (NXT) is a Chinese Materia Medica standardized product extracted from 16 Chinese traditional medical herbs and widely used for treatment of patients with cerebrovascular and cardiovascular diseases in China. Formation of microaneurysms plays an important role in the development of diabetic retinopathy. In this study, we investigated if  NXT can protect diabetic mice against the development of diabetic retinopathy. The *db*/*db* mice (~6 weeks old), a diabetic animal model, were divided into two groups and fed normal chow or plus NXT for 14 weeks. During the treatment, fasting blood glucose levels were monthly determined. After treatment, retinas were collected to determine retinal thickness, accumulation of carbohydrate macromolecules, and caspase-3 (CAS-3) expression. Our results demonstrate that administration of NXT decreased fasting blood glucose levels. Associated with the decreased glucose levels, NXT blocked the diabetes-induced shrink of multiple layers, such as photoreceptor layer and outer nuclear/plexiform layers, in the retina. NXT also inhibited the diabetes-induced expression of CAS-3 protein and mRNA, MMP-2/9 and TNF*α* mRNA, accumulation of carbohydrate macromolecules, and formation of acellular capillaries in the retina. Taken together, our study shows that NXT can inhibit the development of diabetic retinopathy and suggests a new potential application of NXT in clinic.

## 1. Introduction

Diabetes is a big public health problem because it can induce multiple complications in different organs. The number of diabetic patients is expected to be 552 million by 2030 globally [1]. Diabetic retinopathy, one of the most common microvascular complications of diabetes, is a leading cause of vision impairment and blindness in adults [2–4]. Nearly all the patients with type 1 diabetes and more than half of the patients with a 20-year history of type 2 diabetes can develop retinopathy [5]. The development of diabetic retinopathy can be regulated by multiple factors, such as hyperglycemia, oxidative stress, proinflammation, and generation of advanced glycation end products (AGEs) [6–9]. These pathological processes can lead to loss of retinal capillary cells, disruption of vascular barrier, formation of microaneurysms, and preretinal neovascularization [2, 10].

Hyperglycemia plays a central role in the initiation of diabetic retinopathy because it substantially induces pathological changes in the retinal vascular. The epidemiological studies on diabetes demonstrate a strong link between the degree of hyperglycemia and the progression of diabetic retinopathy. Accordingly, lowering plasma glucose levels significantly reduces the prevalence of retinopathy in the diabetic patients. Therefore, the timely tight control of blood glucose is an effective way to reduce the development of diabetic retinopathy [11, 12].

Buchang NaoXinTong capsule (NXT) is an approved traditional Chinese medicine and is used to treat patients with stroke and other vascular diseases. NXT contains the following 16 various kinds of traditional Chinese medicines:* Astragalus membranaceus*,* Salvia miltiorrhiza*,* Ligusticum*, Radix Paeoniae Rubra, Szechwan Lovage Rhizome, Semen Persicae,* Carthamus tinctorius* L., Frankincense, myrrh,* Spatholobus suberectus*, Achyranthes Root, CassiaTwig, Mulberry Twig, earthworms, scorpions, and* Hirudo* [13]. Studies with animal models demonstrate that NXT can protect proatherogenic mice against the development of atherosclerosis by ameliorating serum lipid profiles and inhibiting maturation of dendritic cells [14]. NXT also increases the catalytic activity of the drug metabolizing CYP2C19 enzyme. The combined NXT and clopidogrel further increase the antiplatelet effect of clopidogrel in patients with CYP2C19∗2 gene mutation [15]. All the above observations suggest that NXT has protective effects in cardiac and vascular diseases. Formation of diabetic retinopathy is associated with the pathological progress of microvascular system. Therefore, in this study, we determined if NXT can reduce diabetic retinopathy in an animal model.

## 2. Materials and Methods

### 2.1. Materials

NXT was kindly provided by Xianyang Buchang Pharmaceutical Co. Ltd. (Shan'xi, China). Rabbit anti-CAS-3 polyclonal antibody was purchased from Santa Cruz Biotechnology (Dallas, Texas). All other chemicals were purchased from Sigma-Aldrich (St. Louis, MO) except as indicated.

### 2.2. Animals

The protocol for* in vivo* study with mice was granted by the Committee on the Ethics of Animal Experiments of Nankai University (Tianjin, China) and conforms to the Guide for the Care and Use of Laboratory Animals published by NIH. Both male type 2 diabetic (BKS.C g-m +/+ Lepr^*db*^/J, *db*/*db*) mice and C57BLKS/J wild type mice at the age of 6 weeks were purchased from the Animal Center of Nanjing University (Nanjing, China). The animals were maintained at the Animal Center of Nankai University with free access to food and drinking water.

Based on the clinical usage, the dose of NXT applied to mice was converted into 624 mg/kg body weight/day (mpk) [14]. The male *db*/*db* mice were randomly divided into two groups (10/group) and received following treatment: group 1, mice were fed normal chow; group 2, mice were fed the chow containing NXT (624 mpk). Meanwhile, male C57BLKS/J wild type mice were used as a nondiabetic or normal control. The treatment was continued for ~14 weeks.

### 2.3. Determination of Fasting Blood Glucose Levels

During the treatment, blood was withdrawn from mouse tail vein after overnight fasting at the different time points. Blood glucose levels were determined with a OneTouch glucometer and test strips (LifeScan, Milpitas, CA) according to the manufacture's instruction.

### 2.4. Preparation and PAS Staining of Retinal Vasculature and Quantitation of Acellular Capillaries

Retinal vasculature was prepared based on the method as described [16] with minor modifications. Briefly, mouse eyes were fixed in 4% paraformaldehyde freshly made in PBS (PFA/PBS) overnight after enucleation. The retinas were dissected from eyeballs, washed in water overnight with gentle shaking at room temperature (RT), and then digested in 3% trypsin solution (Invitrogen, Grand Island, NY) for 2-3 h at 37°C. The tissue was then transferred into filtered water and the network of vessels was freed from adherent retinal tissue by gentle shaking and manipulation under a dissection microscope. The vessels were then mounted on clean slides, air-dried completely, and stained with periodic acid Schiff (PAS) solution according to the instruction manual of the manufacture. After the tissue was stained and washed in water, it was then dehydrated and mounted (Permount mounting medium, Fisher Scientific, Pittsburgh, PA). The prepared retinal vessels were observed and photographed under a microscope. The density of PAS staining was quantified with the Photoshop software.

Acellular capillaries were randomly counted with 4–6 filed areas around the midretina. Acellular capillaries were defined as capillary sized vessel tubes with no nuclei along their length [17]. Data are presented as number of acellular capillaries per 10 mm^2^ of retina.

### 2.5. Preparation of Retina Cross Sections and Evaluation of Retinal Capillary Basement Membrane

To evaluate the retinal capillary basement membrane, mouse eyes were fixed in 4% PFA/PBS at 4°C for 12 h followed by cryoprotection in 30% sucrose/PBS overnight before the quick freezing in OCT compound (Sakura Finetek, Inc., Torrance, CA). The 5 *μ*m frozen cross sections were prepared by a standard procedure. The sections were then stained with hematoxylin and eosin (HE) for evaluation of retinal capillary basement membrane. After being stained, the cross sections were observed and photographed under a microscope.

### 2.6. Determination of CAS-3 Protein Expression in Mouse Retina

The above cross sections were used to determine expression of caspase-3 (CAS-3) protein by immunofluorescent staining as follows: the cross sections on cover slides were incubated with rabbit anti-CAS-3 polyclonal antibody overnight at 4°C. After removal of the primary antibody by washing with PBS, the slides were stained with rhodamine-conjugated goat anti-rabbit IgG for 2 h at RT. After being washed with PBS, the slides were restained with DAPI solution for nuclei. Images of all the slides were observed and photographed under a fluorescence microscope.

### 2.7. RNA Isolation and Determination of CAS-3, MMP-9, MMP-2, and TNF*α* mRNA Expression in Mouse Retina

After treatment, mice retinas were removed and homogenized in Trizol reagent (Invitrogen, Carlsbad, CA) to extract total RNA as described [18]. The cDNA was synthesized with the first-stand cDNA synthesis Kit from Fermentas (Pittsburgh, PA). Expression of CAS-3, matrix metalloprotein 2 (MMP-2), MMP-9, and tumor necrosis factor *α* (TNF*α*) mRNA was determined by real time RT-PCR using a SYBR green PCR master mix from Bio-Rad (Los Angeles, CA) and the primers listed in Table 1 and normalized by *β*-actin mRNA in the corresponding samples.

### 2.8. Data Analysis

**Figure 1:**
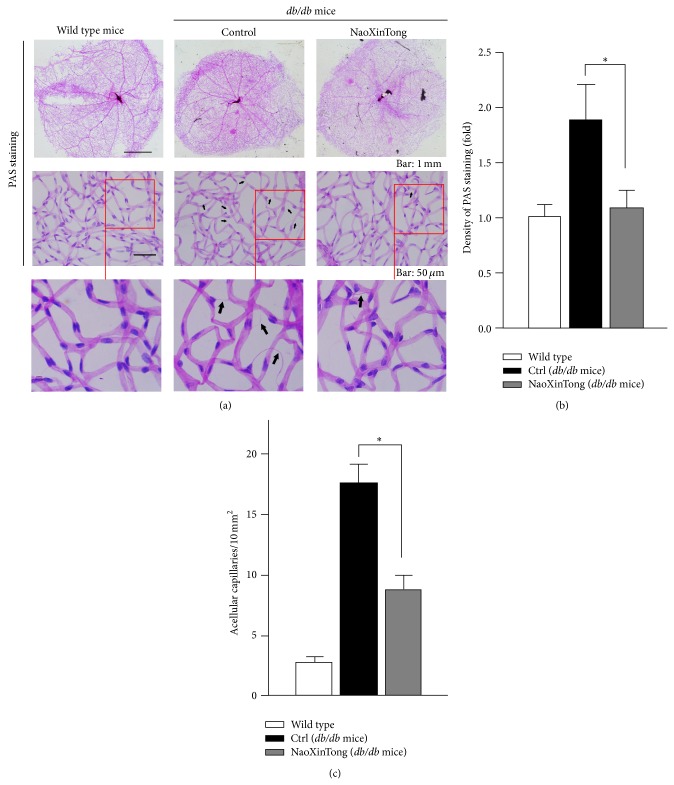
NXT inhibits the accumulation of carbohydrate macromolecules and the formation of acellular capillaries in retinal vasculature in *db*/*db* mice. (a) At the end of the study, mouse eyes were collected and the retinal vascular network was prepared followed by PAS staining and photograph under a microscope as described in Section 2. The representative images from each group were presented. Black arrows indicate acellular capillaries in the retinal vasculature. Bars: 1 mm and 50 *μ*m in the up and middle panels, respectively. (b) The density of PAS staining was quantified by the Photoshop software. (c) Quantitation of acellular capillaries in the retina. ^∗^ *P* < 0.05 (*n* ≥ 3).

All experiments were repeated at least three times, and the representative results are presented. Data in Table 2 and Figures 1, 3, and 4 were presented as mean ± standard error and analyzed by Student's *t*-test (*n* ≥ 3). The differences were considered significant at *P* < 0.05.

## 3. Results

### 3.1. NXT Decreases the Fasting Blood Glucose Levels in *db*/*db* Mice

To determine the effect of NXT on fasting blood glucose levels, during the treatment, the blood samples were monthly collected followed by determination of glucose levels. The results in Table 2 show the low and constant blood glucose levels in wild type mice. In contrast, a higher blood glucose level was seen at the beginning of the study in *db*/*db* mice than wild type mice. More importantly, the higher blood glucose levels kept increasing in control *db*/*db* mice with time. At the end of the study, more than twofold increase (12.3 ± 1.61 versus 29.56 ± 3.43 mM) was determined in control *db*/*db* mice. However, although the administration of NXT to *db*/*db* mice had little effect on blood glucose levels in the first month of treatment, it substantially reduced blood glucose levels thereafter. At the end of the study (~3 months), the blood glucose levels in *db*/*db* mice receiving NXT treatment were reduced to ~60% of control *db*/*db* mice suggesting NXT improves blood glucose levels.

### 3.2. NXT Inhibits the Diabetes-Induced Retinal Vascular Abnormalities

The improvement of blood glucose levels in *db*/*db* mice implies that NXT may prevent the animals from the diabetes-induced retinal vascular abnormalities. To determine it, we initially isolated retinal network of vessels and conducted the PAS staining to assess the effect of NXT on retinal vasculature as well as the accumulation of carbohydrate macromolecules. Compared to wild type mice, the results in Figure 1(a) show a denser image in control *db*/*db* mice than wild type mice which suggests a severe accumulation of carbohydrate macromolecules in the retinal vasculature (up middle panel). Furthermore, the enlarged image displays formation of numerous acellular capillaries in the retinas of control *db*/*db* mice (middle column of Figure 1(a), indicated by the black arrows). However, administration of NXT to *db*/*db* mice significantly decreased the density of the vascular vessels after PAS staining that indicates NXT can prevent the accumulation of carbohydrate macromolecules (Figure 1(b)). In addition, the formation of acellular capillaries was substantially inhibited by NXT (right column of Figures 1(a) and 1(c)). Thus, the results in Figure 1 indicate that NXT protects *db*/*db* mice against the diabetes-induced retinal vascular abnormalities and prevents the occurrence of diabetic retinopathy.

**Figure 2:**
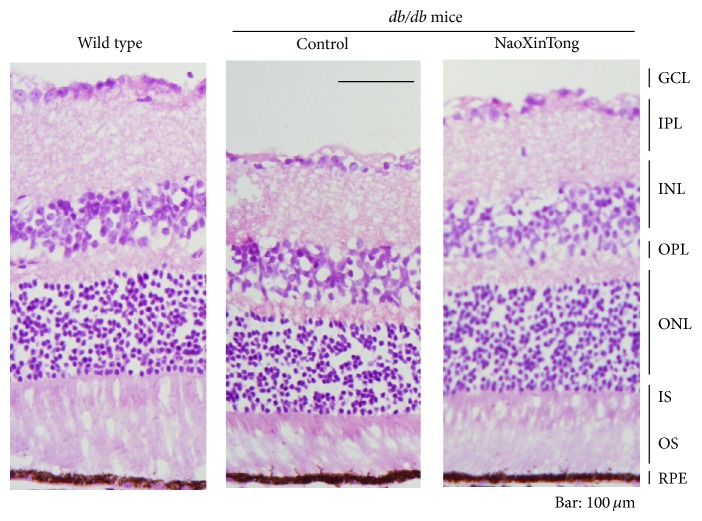
NXT corrects the retinal abnormalities in *db*/*db* mice. Mouse eyeballs were collected at the end of the study and fixed in 4% PFA/PBS. The 5 *μ*m frozen cross sections were prepared and used to conduct HE staining as described in Section 2. The representative images from each group were presented. Bar: 100 *μ*m.

Diabetic retinopathy causes shrink of whole retina which is contributed by shrink of sublayers in the retina. To further determine the effect of NXT on the development of diabetic retinopathy, we collected mouse eyeballs and determined the retinal thickness and structural alterations. The central retinal thickness is defined as the distance from the ganglion cell layer (GCL) to the retinal pigment epithelium layer (RPE) in the central area of retina. The results in Figure 2 show that the whole central retinal thickness in control *db*/*db* mice was significantly reduced when compared to wild type mice. Interestingly, administration of *db*/*db* mice with NXT significantly restored the whole retinal thickness to normal suggesting NXT prevents *db*/*db* mice from the development of diabetic retinopathy.

**Figure 3:**
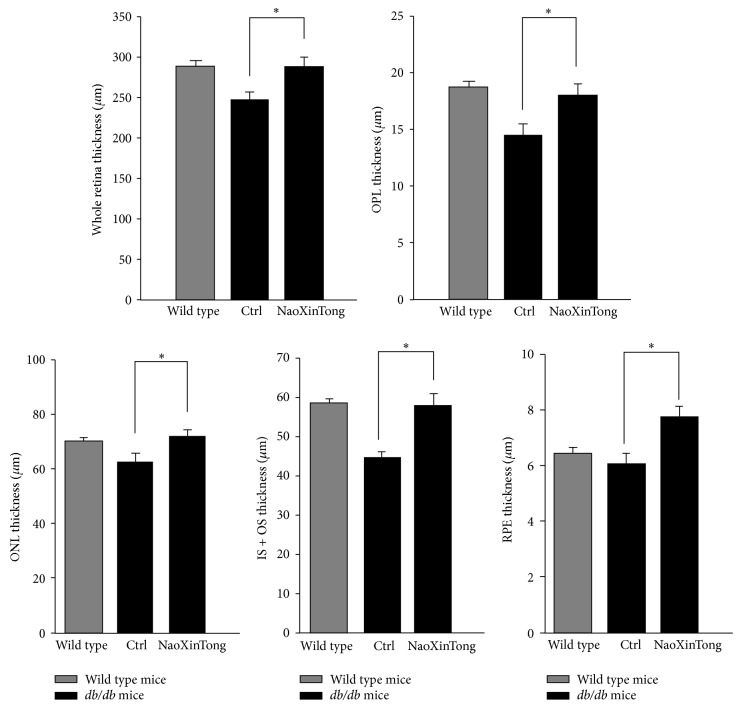
NXT prevents the reduction of thickness of whole retina and sublayers in retina in *db*/*db* mice. After HE staining and photographing, the thickness of whole retina and sublayers in retina was quantified. OPL: the outer plexiform layer; ONL: outer nuclear layer, IS + OS: photoreceptor layer; RPE: retinal pigment epithelium layer. ^∗^ *P* < 0.05 (*n* ≥ 5).

Furthermore, we quantified the thickness of whole retina as well as each sublayer in the retina, such as outer plexiform layer (OPL), outer nuclear layer (ONL), photoreceptor layer (IS + OS), and RPE (Figure 3). Compared to wild type mice, the thickness of OPL, ONL, and the photoreceptor layer (IS + OS) in control *db*/*db* mice was significantly reduced mostly in photoreceptor layer. The combined reduction of thickness in these sublayers contributes to the reduction of whole retinal thickness. In contrast, treatment of *db*/*db* mice with NXT prevented the reduction of the thickness of whole retina, OPL, ONL, and photoreceptor layers.

### 3.3. NXT Inhibits Retinal CAS-3, MMP-2, MMP-9, and TNF*α* Expressions in *db*/*db* Mice

**Figure 4:**
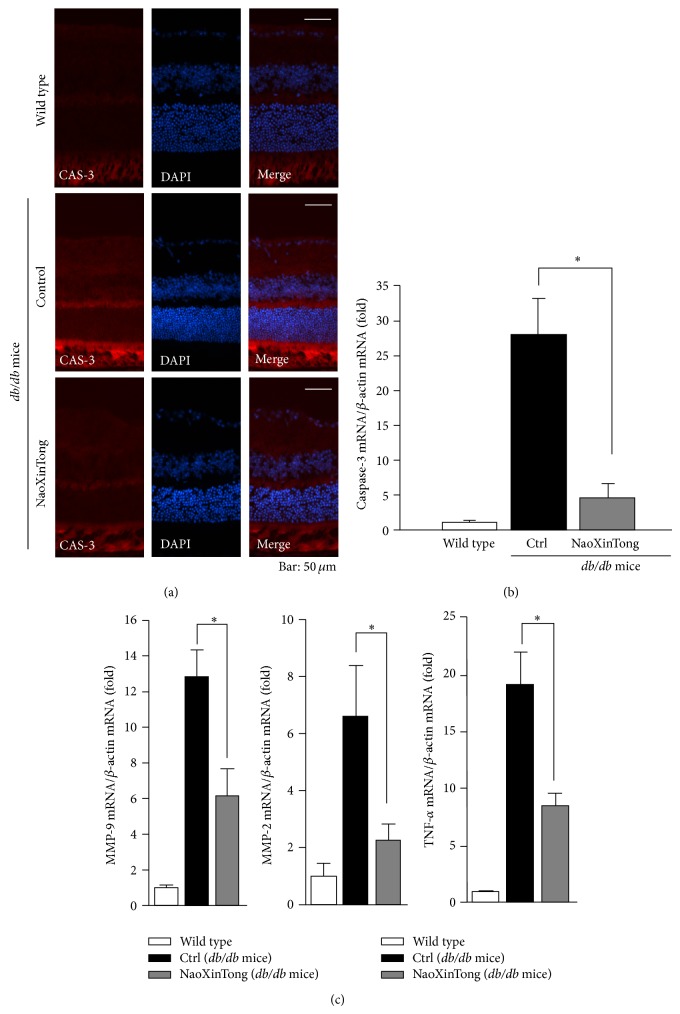
NXT inhibits diabetes-induced CAS-3 expression. (a) The frozen sections of mouse eyeballs from each group were prepared and CAS-3 protein expression was determined by immunofluorescent staining as described in Section 2. Bars: 50 *μ*m. (b) CAS-3 mRNA expression in the retinas was determined by real time RT-PCR analysis. (c) TNF-*α*, MMP-2 and MMP-9 mRNA expression in the retinas was determined by real time RT-PCR analysis. ^∗^ *P* < 0.05 (*n* ≥ 5).

The loss of pericytes in the photoreceptor layer is a determinant of diabetic retinopathy at the early stage in humans and animal models [19]. High glucose levels decrease glutathione levels in pericytes that will activate CAS-3 expression and apoptosis of pericytes [19]. To determine if the protection of retinal structure by NXT is related to regulation of CAS-3 expression, we assessed CAS-3 protein levels by immunofluorescent staining. CAS-3 is mainly expressed in the photoreceptor layer. Compared to wild type mice, the results in Figure 4(a) show that CAS-3 expression was increased in the retina of *db*/*db* control mice, in particular in the photoreceptor layer. However, the increase was inhibited by NXT. Associated with changes of CAS-3 protein, diabetes substantially increased CAS-3 mRNA expression which was also significantly decreased by NXT treatment (Figure 4(b)).

Diabetes can also induce expression of TNF-*α*, MMP-2, and MMP-9 in the retina which may enhance apoptosis in the tissue and contribute to the pathogenesis of diabetic retinopathy [20–22]. To determine if NXT can affect TNF-*α*, MMP-2, and MMP-9 expressions, we assessed mRNA levels of these molecules by real time RT-PCR analysis. Compared to control *db*/*db* mice, Figure 4(c) shows that NXT treatment significantly inhibited TNF-*α*, MMP-2, and MMP-9 mRNA expressions in the retinas of *db*/*db* mice. Taken together, the results in Figure 4 indicate that NXT can inhibit the diabetes-induced apoptosis in retina and protect the retinal normal structure and physiological function by reducing production of inflammation as well as apoptosis.

## 4. Discussion

NXT has been demonstrated to protect patients with cardiac and vascular diseases. The diabetic retinopathy is a prevalent and profound microvascular disease in diabetic patients. The patients with diabetic retinopathy are 25-fold more likely to be blind than normal individuals [5] while the diabetic retinopathy is the leading cause of blindness in working age adults worldwide [3, 23]. In this study, we demonstrate the antidiabetic retinopathy properties of NXT. Our study shows that NXT prevents the formation of acellular capillaries and accumulation of carbohydrate macromolecules and inhibits the shrink of retina and retinal sublayers which is associated with reduction of CAS-3 and some inflammatory molecules expression in the retina. Taken together, NXT well maintains structural integrity of retina in *db*/*db* mice and inhibits the development of diabetic retinopathy.

Mounting evidence has supported that hyperglycemia can promote the development of diabetic retinopathy [24–26]. High glucose levels decrease glutathione level in pericytes that can result in mitochondrial overproduction of reactive oxygen species (ROS) in the diabetic microvasculature [27]. In turn, the increased ROS activates diacylglycerol- (DAG-) PKC pathway to induce expression of vascular endothelial growth factor (VEGF) and generation of AGEs. The combination of these effects accelerates the loss of pericytes followed by degeneration of endothelial cells and capillary occlusions [28, 29]. Therefore, lowering plasma glucose levels is believed to be an effective way to reduce the development of diabetic retinopathy. In this study, we determined that NXT reduces fasting blood glucose levels, indicating NXT inhibits diabetic retinopathy which may partially be through the control of glucose levels. Moreover, we observed that the induction of retinal CAS-3, TNF-*α*, MMP-2, and MMP-9 expressions by diabetes was inhibited by NXT treatment, suggesting an antiapoptotic and anti-inflammatory effects of NXT.

In conclusion, our study indicates that NXT inhibits the development of diabetic retinopathy in *db*/*db* mice and implies an important and potential application of NXT for treatment of diabetic retinopathy in clinics.

## Figures and Tables

**Table 1:** Sequences of the primers for real time RT-PCR analysis.

Gene	Forward	Backward
CAS-3	GACTTGCTCCCATGTATGGTC	ATCAAAGCGCAGTGTCCTG
MMP-2	TGGCAAGGTGTGGTGTGCGAC	TCGGGGCCATCAGAGCTCCAG
MMP-9	GGTGTGCCCTGGAACTCACACG	AGGGCACTGCAGGAGGTCGT
TNF*α*	GTTCTATGGCCCAGACCCTCAC	GGCACCACTAGTTGGTTGTCTTTG
*β*-actin	ATCTGGCACCACACCTTC	AGCCAGGTCCAGACGCA

**Table 2:** NXT reduces the fasting blood glucose levels in *db/db* mice.

Group	Time of treatment (days)			
	0	31	66	96
*db/db* mice (control)	12.30 ± 1.61	15.29 ± 1.34	26.68 ± 0.84	29.56 ± 1.09
*db/db* mice (NXT)	12.60 ± 2.00	16.87 ± 1.93	21.06 ± 2.06^*^	20.48 ± 1.52^*^
Wild type mice	6.78 ± 0.59	5.96 ± 0.18	5.70 ± 0.16	6.34 ± 0.22

Male *db/db* mice (~6 weeks old) were randomly divided into two groups (10/group) and received the following treatment: group 1 (control), mice were fed normal chow; group 2 (NXT), mice were fed the chow containing NXT (624 mpk) for ~14 weeks. Wild type mice on normal chow were used as nondiabetic or normal control. The blood samples were collected at the indicated time points for determination of blood glucose levels as described in Section 2. ^*^Significantly different from control *db/db* mice at *P* < 0.05 (*n* = 10).
